# Virulence factors of *Mycoplasma synoviae*: Three genes influencing colonization, immunogenicity, and transmissibility

**DOI:** 10.3389/fmicb.2022.1042212

**Published:** 2022-11-25

**Authors:** Sara M. Klose, Oluwadamilola S. Omotainse, Sahar Zare, Paola K. Vaz, Parisa Armat, Pollob Shil, Nadeeka Wawegama, Anna Kanci Condello, Denise O'Rourke, Jillian F. Disint, Daniel M. Andrews, Gregory J. Underwood, Chris J. Morrow, Marc S. Marenda, Amir H. Noormohammadi

**Affiliations:** ^1^Asia-Pacific Centre for Animal Health, Faculty of Veterinary and Agricultural Sciences, Melbourne Veterinary School, The University of Melbourne, Werribee, VIC, Australia; ^2^Asia-Pacific Centre for Animal Health, Faculty of Veterinary and Agricultural Sciences, Melbourne Veterinary School, The University of Melbourne, Parkville, VIC, Australia; ^3^Bioproperties Pty Ltd., Ringwood, VIC, Australia

**Keywords:** *Mycoplasma*, live vaccine, pathogenicity, immunogenicity, temperature sensitivity

## Abstract

Infections caused by *Mycoplasma synoviae* are major welfare and economic concerns in poultry industries worldwide. These infections cause chronic respiratory disease and/or synovitis in chickens and turkeys leading to reduced production and increased mortality rates. The live attenuated vaccine strain MS-H (Vaxsafe^®^ MS), commonly used for protection against *M. synoviae* infection in many countries, contains 32 single nucleotide variations compared to its wildtype parent strain, 86079/7NS. Genomic analysis of vaccine strains reisolated from flocks following the administration of MS-H has identified reversions to the original 86079/7NS sequence in the *obgE*, *oppF* and *gapdh* genes. Here, three MS-H field reisolates containing the 86079/7NS genotype in *obgE* (AS2), *obgE* and *oppF* (AB1), and *obgE*, *oppF* and *gapdh* (TS4), as well as the vaccine MS-H and the parental strain 86079/7NS were experimentally inoculated to chickens. The strains were assessed for their ability to infect and elicit immune responses in the recipient chickens, as well as in naïve in-contact chickens. Despite the loss of temperature sensitivity phenotype and colonization of the reisolates in the lower respiratory tract, there was no significant differences detected in the microscopic mucosal thickness of the middle or lower trachea of the inoculated chickens. Concurrent reversions in ObgE, OppF and GAPDH proteins were associated with higher gross air sac lesion scores and increased microscopic upper-tracheal mucosal thickness in chickens directly inoculated with the reisolates following intratracheal administration of a virulent strain of infectious bronchitis virus. The gross air sac lesions of the chickens in-contact with those inoculated with reisolates were not significantly different to those of chickens in-contact with MS-H inoculated chickens, suggesting that horizontal transmission of the reisolates in the poultry flock will not lead to higher pathogenicity or clinical signs. These results suggest a significant role of GAPDH and/or cumulative effect of ObgE, OppF and GAPDH on *M. synoviae* pathogenicity. Future experiments will be required to investigate the effect of single mutations in *gapdh* or *oppF* gene on pathogenicity of *M. synoviae*.

## Introduction

*Mycoplasma synoviae* is an important pathogen of commercial poultry affecting multiple organs in chickens and turkeys and imposing welfare concerns due to respiratory illness and lameness (Ferguson-Noel et al., 2020; Yadav et al., 2021). The infections caused by this bacterium are responsible for significant economic loss to the intensive poultry industry due to reduced egg production and downgrading of egg quality (Nascimento et al., 2005; Feberwee et al., 2009). The Vaxsafe^®^ MS (MS-H) vaccine was developed by chemical mutagenesis of the *M. synoviae* field strain 86079/7NS (Morrow et al., 1998). While the virulent strain 86079/7NS, hereafter referred to as 7NS, can grow at temperatures up to 39.5°C, the MS-H strain is temperature-sensitive (*ts^+^*) and unable to grow optimally at 39.5°C (Morrow et al., 1998). This property is the basis for the phenotypic assessment of *M. synoviae* field isolates obtained from vaccinated flocks. It is hypothesized that *ts^+^* mycoplasma vaccines colonize the upper respiratory tract, which has a lower temperature than the core body temperature, and induce an immune response without invading or causing inflammation in the lower respiratory system which is at a higher temperature (Whithear et al., 1990). However, occasional detection of *ts^−^ MS*-H reisolates from vaccinated chickens in the absence of disease suggested that attenuation of MS-H is not exclusively due to its *ts^+^* phenotype (Morrow, 1991; Noormohammadi et al., 2003; Nicholas et al., 2009). The discovery of 32 single nucleotide polymorphisms (SNPs) between the genomes of MS-H and its parent strain (Zhu et al., 2019) has given insights on the basis of virulence attenuation in the vaccine strain. Unusually high systemic antibody responses to *M. synoviae* in vaccinated flocks have been reported, and several MS-H derivatives have been isolated from these farms. The genomes of these reisolates occasionally carry reversions to the 7NS genotype in the genes *obgE*, *oppF* and *gapdh*, along with diverse repertoires of other sequence variations (Kordafshari et al., 2020). These observations raised the question of the genetic, phenotypic, and immunogenic stability of MS-H in field conditions. In the present study, three *M. synoviae* isolates recovered from vaccinated flocks were sequenced entirely and their genomes were compared to those of strains 7NS and MS-H. This analysis identified three loci carrying reversions to the 7NS genotype, while the rest of the genome retained the MS-H sequence. One isolate contained a single reversion in the *obgE* gene, another isolate carried an additional reversion in the *oppF* gene, and the last isolate had an additional reversion in the *gapdh* gene, resulting in three different genotypes representing a progressive accumulation of these three sequence variations. To evaluate the effect of these sequence variations *in vivo*, and to provide insight into the behavior of the live vaccine in poultry farms, specific pathogen free (SPF) chickens were experimentally infected with each of the three MS-H reisolates carrying variation(s) in these loci. The inoculated and in-contact chickens were monitored for the presence of live *M. synoviae*, lesions, and seroconversion.

## Materials and methods

### Culture medium

The *M. synoviae* cultures were grown in mycoplasma broth (MB) containing 10% swine serum (Sigma-Australia) and 0.01% nicotinamide adenine dinucleotide (Sigma-Australia) based on the formulation of Frey’s medium with minor modification (Frey, 1968; Jones et al., 2006). The cultures were incubated at 37°C until late logarithmic phase (approximately pH 6.8; Shahid et al., 2013a).

### Mycoplasma strains

The wild-type parent 7NS strain was available in our laboratory and its origin has been described previously (Morrow, 1991). The MS-H working seed used in commercial production of the Vaxsafe^®^ MS vaccine, was provided by Bioproperties, Pty. Ltd, Melbourne, Australia. The AS2, AB1, and TS4 reisolates were collected from the choanal cleft of commercial layer chickens from different Australian poultry farms, 11, 28, and 28 weeks after vaccination with Vaxsafe^®^ MS, respectively (Jones, 2007). For DNA extraction and whole genome sequencing, cultures were thawed and 200 uL were inoculated in 40 mL MB and incubated at 37°C until late logarithmic phase

### Illumina sequencing and variation assessment

An aliquot of each reisolate was grown in 40 mL MB and incubated at 37°C until late logarithmic phase. Cells were collected from the culture by centrifugation at 10,000 ×*g* for 20 min at 4°C followed by two steps of washing with 1 mL PBS. The DNA was extracted using Qiagen’s DNeasy Blood and tissue kit and the concentration and quality of the extracted DNA was determined using Qubit and Nanodrop, respectively. In the next step, 100 ng of extracted DNA was used to prepare sequencing libraries using Illumina’s Nextera Flex DNA library prep kit. Sequencing was performed on the Miseq platforms using paired end 300 bp reads at the Charles River Laboratories, Victoria, Australia. The Illumina short-reads were processed using Trim Galore v0.6.6 (Krueger, 2015) to trim bases below Phred quality 25 and remove the Nextera adapter sequences, followed by confirmation of the quality of the filtered reads using FastQC v0.11.9 (Andrews, 2010). The reads were aligned to the MS-H sequence (Genbank accession number CP021129) for SNP and insertion/deletion (indel) analyses using Snippy v4.6.0 (Seemann, 2015). The key parameters of variant calling by snippy included a minimum number of 10 reads coverage to consider variant calling, and at least 5% of those reads differing from the reference to call a variation. The highly repetitive, variable, and similar regions of the genomes such as the *vlhA* gene and pseudogenes, IS1634 family transposase, and type III restriction endonuclease subunit M regions were excluded from variation assessment.

### Nanopore sequencing and genome assembly

The cells were collected and washed as described above. The DNA was extracted using Promega Wizard^®^ HMW DNA Purification Kit, the concentration and quality of the extracted DNA was determined using Qubit and Implen, respectively. In the next step, 400 ng of extracted DNA was used to prepare sequencing libraries using Oxford Nanopore Technology (ONT) Rapid Barcoding (SQK-RBK004) kit. Sequencing was performed on a MinION MK-I device fitted with a FLO-MIN106 flow cell (R9.5 chemistry) and processed using the ONT high accuracy Guppy basecaller v6.1.7. The Nanopore reads were processed using Guppy barcoder v6.1.7 to remove the barcodes and then filtered using NanoFilt v2.6.0 to include only the reads with a minimum length of 1000 bp and quality score of 12 (De Coster et al., 2018). The filtered Nanopore reads were *de novo* assembled using Flye v2.9 (Kolmogorov et al., 2019). The long-read assembly was then polished based on the Illumina short reads using Polypolish v0.5.0 (Wick and Holt, 2022). The polished long-read assembly was used alongside with the long and short reads for hybrid *de novo* assembly using Unicycler v0.4.9b (Wick et al., 2017). To confirm the accuracy of the assembly, short and long reads were mapped to the assembled genome using Geneious Mapper with medium sensitivity in Geneious Prime v2021.1.1 software.

### Titration of the cultures used in chicken inoculation

The strains/reisolates were recovered from −70°C by 1:20 dilution in fresh MB medium and incubated at 37°C for 18 h, followed by a 1:10 dilution in fresh MB medium to make a final volume of 100 mL. The cultures were incubated at 37°C until reaching the mid-logarithmic phase of growth. The starting cultures were diluted in a final volume of 100 mL so that all contained similar titres. An aliquot of each culture was removed prior to aerosolisation to estimate the viable count of the cultures using the most probable number (MPN) method by serial dilution in MB in 96-well plates (Thermo Fisher Scientific). The color change in the medium after 2 weeks of incubation, indicating growth of the bacteria, was used to determine the MPN of mycoplasmas present in each of the inocula (Meynell and Meynell, 1970).

### Temperature sensitivity phenotyping

The temperature-sensitive (*ts^+^*) phenotype of cultures was determined by titration and incubation at permissive (33°C) and non-permissive (39.5°C) temperatures, as described previously (Morrow et al., 1998). Briefly, the strains/reisolates were recovered from-70°C by 1:10 dilution in fresh MB medium and incubated at 33°C for 18 h, followed by serial dilution in MB in four 96-well plates (Thermo Fisher Scientific), and incubation of duplicate trays at 33°C and 39.5°C for 2 weeks. The titres of strains/reisolates at different temperature was determined as described above. Temperature sensitive phenotype was defined by observation of decrease in titre of more than 10^3^ CCU/mL when grown at 39.5°C compared to that of 33°C (Nonomura and Imada, 1982).

### Inoculation and exposure of chickens

**Figure 1:**
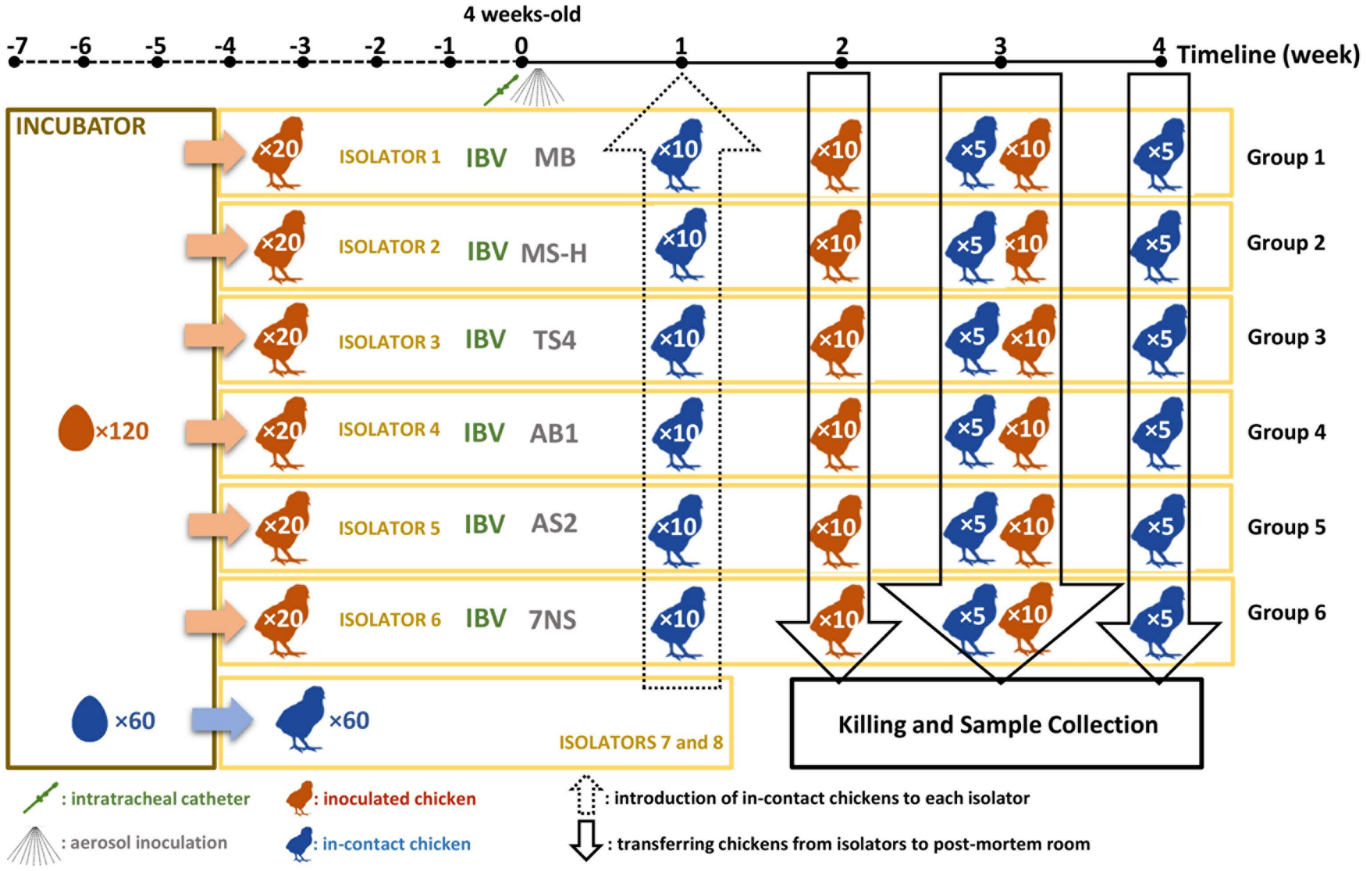
Schematic figure of the chicken experiment. A total number of 180 specific pathogen free (SPF) chickens were hatched. A total of 120 chickens (test chickens) were randomly divided into 6 isolators, while 60 in-contact chickens were randomly allocated into 3 separate isolators. At the age of 4-week, the test chickens inoculated with test substances. One week later, the in-contact chickens were moved into the test isolators (10 chickens per isolator). A total number of 60 chickens (10 from each group) will then be humanely killed 2 weeks later. After one more week, the rest of the test chickens (10 per group) as well as 5 in-contact chickens per group were humanely killed. One week later, the rest of the in-contact chickens (5 per group) were humanely killed.

All procedures involving animals were reviewed and approved by the University of Melbourne Animal Ethics Committee under approval number 21064. A total of 120 four-week-old SPF chickens were hatched from eggs supplied by Australian SPF Services (Woodend, Victoria, Australia) at the animal experimentation facilities of the Asia-Pacific Centre for Animal Health (APCAH). They were randomly allocated into six groups of 20 chickens and housed in separate High Efficiency Particulate Air (HEPA) filtered isolator unit under negative pressure, with feed and water provided *ad libitum* (Figure 1). The *M. synoviae* cultures were grown *in vitro* to mid-logarithmic phase prior to aerosolisation as described above. A virulent Australian field strain of infectious bronchitis virus (IBV) strain V1/71 (Ignjatovic et al., 2002), was administered intratracheally (7.9 × 10^3.5^ EID50/mL in a final volume of 200 μL per chicken) to chickens immediately prior to inoculation with each *M. synoviae* strains/reisolates as described previously (Markham et al., 1998). The chickens were inoculated with 32 to 36 mL of MS-H, AS2, AB1, TS4 and 7NS strain containing 1.18 to 3.66 × 10^7^ color changing units (CCU) per milliliter (Supplementary Table S2) by aerosolisation of 100 mL cultures using compressed air into a purpose-built infection chamber for 40 min (Kanci et al., 2017). One group of chickens was inoculated with sterile MB media by aerosol and IBV by intratracheal route, as a negative *M. synoviae* control. One week after inoculation, 10 uninoculated (in-contact) 5-week-old SPF chickens were placed into each isolator (Figure 1).

### Assessment of pathogenicity

Ten randomly selected inoculated chickens from each group were humanely killed and necropsied at 6 weeks of age (2 weeks post inoculation; WPI), and the remaining ten inoculated chickens were humanely killed and necropsied at 7 weeks of age (3 WPI). Five randomly selected in-contact chickens were humanely killed and necropsied at 7 weeks of age (2 weeks post exposure, WPE), and the remaining five test chickens were humanely killed and necropsied at 7 weeks of age (3 WPE). Thoracic and abdominal air sac lesions were scored grossly for severity as described previously (Noormohammadi et al., 2003). A cumulative lesion score for each chicken was determined by combining the individual scores given to each anatomically distinct air sac on a scale of 0–3 (with a maximum score of 18 for a chicken if all six air sacs were given a score of 3). The overall lesion scores were compared using the Dunn’s corrected Kruskal–Wallis test, with *P <* 0.01 considered significant. The tissues collected from upper, middle, and lower trachea were processed and stained with hematoxylin and eosin for histopathology examination as described previously (Omotainse et al., 2022). The tracheal mucosal thickness was measured at upper, middle, and lower trachea using a graticule lens at a magnification of 400X at six equidistant points. An average thickness was calculated from these three levels as well. The Tukey’s corrected 2-way ANOVA test was used to compare the thickness of tracheal mucosa with *P <* 0.01 considered significant.

### Serology

Serum samples were obtained from blood clot in heart chambers during post-mortem examination and stored at −20°C for future enzyme-linked immunosorbent assay (ELISA). An in-house recombinant MSPB ELISA was used to measure the systemic antibody responses against the *M. synoviae* strains/reisolates (Noormohammadi et al., 1999). The means of the absorbance obtained from duplicated serum samples were interpolated from a standard curve using a *M. synoviae* positive serum to determine the relative antibody units. The Tukey’s corrected 2-way ANOVA test was used to compare the relative antibody units with *P <* 0.01 considered significant. Three-Sigma limit (three standard deviations from mean) of relative antibody unit values in known negative group was used to calculate a cut-off value. Sera with relative antibody unit values above the cut-off value were considered positive. Two-tailed Fisher’s Exact test was used to compare the seropositivity rates with *P <* 0.01 considered significant.

### Reisolation of *Mycoplasma synoviae* from chickens

Swabs taken from upper trachea, lower trachea, air sacs, spleen or kidney were used to inoculate MB, which was then incubated at 37°C and examined daily for an acidic color change indicative of mycoplasma growth. Colonization rates were determined from the number of cultures showing color change compared to total number of cultures (presented as percentage). Cultures with no color change after 3 weeks incubation were considered as lack of colonization of the strain/reisolate in that anatomic region. Two-tailed Fisher’s Exact test was used to compare the reisolation rates with *P <* 0.01 considered significant.

### Statistical analysis

The statistical analyses for all the experiments were performed using GraphPad Prism (v9.2.0 for Windows). Specific tests used for different criteria have been described in relevant sections above.

## Results

### One to three loci reverted to a parental genotype in MS-H reisolates, while 29/32 sequence variations associated with the vaccine strain were conserved

The genomes of the strains AS2, AB1 and TS4, which were all reisolated from vaccinated poultry farms, were nearly identical to the sequence of the MS-H vaccine. Only three reversions into a 7NS genotype were identified in these reisolates, as follows: (1) A/G SNP at nucleotide position 193771, resulting in amino acid change from Arginine to Glycine at position 123 of the ObgE GTPase coding sequence; (2) T insertion at nucleotide position 397777, resulting in frameshift at position 156 of the OppF ATP-binding cassette (ABC) transporter coding sequence; and (3) A/G SNP at nucleotide position 296526 resulting in amino acid change from Lysine to Arginine at position 306 of the of the Glyceraldehyde-3-phosphate dehydrogenase (GAPDH) coding sequence. The occurrence and distribution of these three reversions to a 7NS genotype were strain-dependent. Reisolate AS2 (GenBank accession number CP103982) contained a variation in the *obgE* gene. Reisolate AB1 (GenBank accession number CP103981) contained variations in the *obgE* and *oppF* genes. Reisolate TS4 (GenBank accession number CP103980) contained variations in the *obgE*, *oppF* and *gapdh* genes (Table 1).

**Table 1:** Comparison of genome sequences of the MS-H reisolates to those of MS-H and 7NS (excluding the repetitive and variable regions).

Nucleotide position*	86079/7NS	UoM_MS-H	AS2	AB1	TS4	Protein/Region	Amino acid change*
14181	G	A	A	A	A	tRNA methyltransferase	-
36932	A	T	T	T	T	Non-coding region	-
61685	G	A	A	A	A	Topoisomerase IV subunit A	-
62874	G	A	A	A	A	Excinuclease ABC subunit B	-
67028	G	A	A	A	A	tRNA-Trp	-
104704	G	A	A	A	A	Non-coding region	-
107765	G	A	A	A	A	Multiple sugar ABC transporter	-
193771	G	A	G	G	G	GTP-binding protein, ObgE	Arg123Gly
201094	G	A	A	A	A	p80-related protein	-
203205	C	A	A	A	A	Non-coding region	-
242451	G	A	A	A	A	Glyceraldehyde-3-phosphate dehydrogenase, GAPDH	-
296526	G	A	A	A	G	Glyceraldehyde-3-phosphate dehydrogenase, GAPDH	Lys306Arg
352952	G	A	A	A	A	VACB-like ribonuclease II	-
389629	G	A	A	A	A	Aspartate-ammonia ligase	-
397777	T	—	—	T	T	ABC transporter, OppF	Asn156fs
433343	G	A	A	A	A	Alanyl-tRNA synthetase	-
438657	G	A	A	A	A	Triacylglycerol lipase	-
481287	G	A	A	A	A	Haemolysin C	-
498421	T	C	C	C	C	Histidyl-tRNA synthetase	-
502827	TA	—	—	—	—	Non-coding region	-
522899	G	A	A	A	A	Hypothetical protein	-
563391	G	A	A	A	A	Hexosephosphate transport protein	-
567729	C	A	A	A	A	DNA-directed RNA polymerase beta	-
584838	G	A	A	A	A	Hypothetical protein	-
615741	G	A	A	A	A	Potassium uptake protein, KtrB	-
628272	G	A	A	A	A	DNA polymerase III alpha	-
679814	G	A	A	A	A	Cation-transporting P-type ATPase	-
686265	G	A	A	A	A	Non-coding region	-
716623	G	A	A	A	A	Hypothetical protein	-
737180	G	A	A	A	A	Hypothetical protein	-
780237	AT	—	—	—	—	Non-coding region	-
794057	G	A	A	A	A	Uridylate kinase	-

*Nucleotide position and amino acid change corresponding to the UoM_MS-H genome and amino acid sequences (Genbank accession number CP021129); fs: frame shift.

### All three MS-H field reisolates had *ts* phenotype consistent with that of 7NS

The viable cell counts of the cultures at permissive (33°C) and non-permissive (39.5°C) temperatures were measured (Table 2). The 7NS, AS2, AB1, TS4 titres at 39.5°C did not show any fold change above 10^1^ CCU/mL when compared to the titres at 33°C, reflecting *ts^−^* phenotype. However, MS-H showed a 2.07 × 10^5^ CCU/mL decline in the titre at 39.5°C compared to 33°C, reflecting its *ts^+^* phenotype.

**Table 2:** Viable counts of *Mycoplasma synoviae* strains/reisolates (CCU/mL) in this study at permissive (33°C) and non-permissive (39.5°C) temperatures.

	33°C	39.5°C	Log change	Phenotype
MS-H	2.4 × 10^7^ (1 × 10^7^)	1.15 × 10^2^ (1.88 × 10^1^)	7.38	*ts^+^*
AS2	4.93 × 10^7^ (3.08 × 10^7^)	3.46 × 10^7^ (1 × 10^7^)	0	*ts^−^*
AB1	6.66 × 10^7^ (0)	2.4 × 10^7^ (1 × 10^7^)	0	*ts^−^*
TS4	7.99 × 10^7^ (1.88 × 10^7^)	2.69 × 10^7^ (5.97 × 10^6^)	0	*ts^−^*
7NS	6.97 × 10^6^ (3.96 × 10^7^)	4.88 × 10^6^ (2.51 × 10^7^)	0	*ts^−^*

Data are presented as means (standard deviation). Log change denotes the difference between the calculated viable counts measured at 33°C and the viable counts measured at 39.5°C. CCU, color changing units. *ts^+^*, temperature-sensitive. *ts^−^*, non-temperature-sensitive.

### In-contact chickens from the groups inoculated with reisolates had high humoral antibody responses

Means and standard deviations of relative antibody units against MSPB at 2- and 3-week post inoculation, and 2- and 3-week post exposure of the in-contact chickens are shown in Supplementary Table S3. All the chickens inoculated with the TS4 reisolate showed significant higher seropositivity compared to the negative control (Table 3). At 3-week post inoculation, the AB1 and 7NS inoculated chickens showed significant higher seropositivity compared to the negative control. The seropositivity of the in-contact chickens after 3-week contact with chickens inoculated with AS2 was significantly higher than the negative control (Table 3).

**Table 3:** Seropositivity of the *M. synoviae* cultures 2- and 3-week after aerosol inoculation of SPF chickens with various strains or reisolates of *M. synoviae* following intratracheal inoculation with a virulent strain of IBV, and 2- and 3-week after exposure of in-contact chickens with the inoculated chickens.

Inoculum	Inoculated chickens		In-contact chickens	
	2 WPI	3 WPI	2 WPI	3 WPI
MB	0/10	0/10	0/5	0/5
MS-H	2/10	0/10	2/5	2/5
AS2	6/10	3/10	4/5	5/5^*^
AB1	6/10	8/10^**^	3/5	2/5
TS4	10/10^***^	10/10^***^	2/5	1/5
7NS	3/10	8/10^**^	3/5	3/5

Data are presented as the numbers of seropositive chickens/the numbers of all the chickens in the same group. Sera with relative antibody unit values above the cut-off value calculated using the Three-Sigma limit (three standard deviations from mean) of relative antibody unit values in the negative control group, were considered positive. *p < 0.01, **p < 0.001, ***p < 0.0001 (two-tailed Fisher’s Exact test). WPI, weeks post inoculation; WPE, weeks post exposure; AS2, MS-H reisolate containing reversion in *obgE* gene; AB1, MS-H reisolate containing reversions in *obgE* and *oppF* genes; TS4, MS-H reisolate containing reversions in *obgE*, *oppF* and *gapdh* genes; MB, mycoplasma broth.

### The MS-H reisolates colonised the trachea of most inoculated chickens

The MS-H strain could not be recovered from the upper trachea, lower trachea, air sacs, spleen or kidney of the MS-H inoculated chickens and the in-contact chickens (Table 4). None of the strains or reisolates were reisolated from kidney or spleen of any of the inoculated chickens. In comparison to MS-H, the strain AS2 was recovered significantly higher from the upper trachea of inoculated chickens. The reisolate AB1 was recovered significantly higher than MS-H from upper trachea of inoculated chickens 3-week post inoculation. At 2-week post inoculation, the TS4 reisolate was recovered significantly higher than MS-H from the upper trachea of inoculated chickens 2-week post inoculation, as well as upper and lower trachea of inoculated chickens 3-week post inoculation.

**Table 4:** Reisolation of the *M. synoviae* strains from upper trachea (UT), lower trachea (LT), or air sacs (AS), 2- and 3-week after aerosol inoculation of SPF chickens with various strains or reisolates of *M. synoviae* following intratracheal inoculation with a virulent strain of IBV, and 2- and 3-week after exposure of in-contact chickens with the inoculated chickens.

Inoculum	Inoculated chickens						In-contact chickens	
	2 WPI			3 WPI			2 WPE	3 WPE
	UT	LT	AS	UT	LT	AS	UT	UT
MB	0/10	0/10	0/10	0/10	0/10	0/10	0/5	0/5
MS-H	0/10	0/10	0/10	0/10	0/10	0/10	0/5	0/5
AS2	7/10^*^	0/10	0/10	80^**^	0/10	0/10	1/5	4/5
AB1	5/10	2/10	1/10	7/10^*^	3/10	0/10	05	2/5
TS4	9/10^**^	5/10	2/10	10/10^***^	7/10^*^	4/10	0/5	2/5
7NS	2/10	1/10	0/10	3/10	0/10	0/10	1/5	3/5

Data are presented as number of cultures showing color change/number of all the cultures in the same group. *p < 0.01, **p < 0.001, ***p < 0.0001 (two-tailed Fisher’s Exact test). WPI, weeks post inoculation; WPE, weeks post exposure; AS2, MS-H reisolate containing reversion in *obgE* gene; AB1, MS-H reisolate containing reversions in *obgE* and *oppF* genes; TS4, MS-H reisolate containing reversions in *obgE*, *oppF* and *gapdh* genes.

### Gross air sac lesion scores of the reisolate in-contact chickens were not significantly different to those of the chickens in-contact with MS-H inoculated chickens

**Figure 2:**
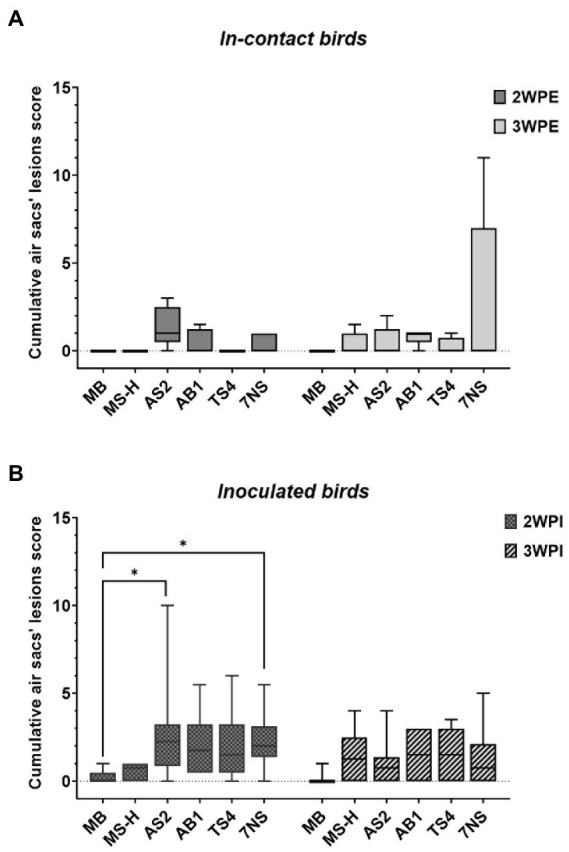
Box plots showing the data range (boxes: upper and lower quartile, whiskers: minimum and maximum) and line indicating the median value of the scores of the gross air sac lesions, 2- and 3-week after exposure of in-contact chickens to inoculated chickens **(A)**, and 2- and 3-week after aerosol inoculation of chickens with various strains or reisolates of *M. synoviae* following intratracheal inoculation of virulent IBV **(B)**. Air sac lesions were scored grossly for severity on a scale of 0–3, and a cumulative score was determined for each chicken by adding the scores of all air sacs. *p < 0.01 (Dunn’s corrected Kruskal–Wallis test). WPI, weeks post inoculation; WPE, weeks post exposure; AS2, MS-H reisolate containing reversion in *obgE* gene; AB1, MS-H reisolate containing reversions in *obgE* and *oppF* genes; TS4, MS-H reisolate containing reversions in *obgE*, *oppF* and *gapdh* genes.

Median and range of cumulative air sac scores for each group are shown in Supplementary Table S1. While there were only minor lesions observed in the in-contact chickens from the groups that received the MS-H vaccine or the farm reisolates, the in-contact chickens exposed to 7NS-inoculated chickens contained more severe gross air sac lesions (range: 0–11) 3-week post exposure (Figure 2). At 2-week post inoculation, the chickens from the groups inoculated with AS2 and 7NS showed air sac lesions with a median score significantly (*p <* 0.01) higher than the negative control group (Figure 2). However, the median air sacs lesion scores of chickens directly inoculated with AS2, AB1, TS4, and 7NS were not significantly different to those of MS-H inoculated chickens 3 weeks after inoculation.

### No significant differences were observed in mucosal thickness of lower or middle trachea of the inoculated chickens

The tracheal mucosal thickness was measured for the chickens inoculated with different strains or reisolates. Means and standard deviations for mucosal thickness for upper, lower, and middle trachea for each group are shown in Table 5. No statistically significant differences were found between groups at lower and middle trachea either 2- or 3-week post inoculation. The mucosal thickness at upper trachea of the chickens inoculated with the TS4 reisolate were significantly (*p <* 0.01) higher than all other groups at 2- and 3-week post inoculation (Supplementary Figure S1). With mucosal thicknesses from three levels of trachea (upper, middle, and lower), an average tracheal thickness was also calculated for each group (Table 5). At 3-week post inoculation, the chickens inoculated with TS4 showed average tracheal thicknesses that were significantly greater than those inoculated with MS-H or MB (*p <* 0.01).

**Table 5:** Mucosal thicknesses of upper trachea (UT), middle trachea (MT), and lower trachea (LT) collected 2- and 3-week after inoculation of chickens with various strains or reisolates of MS following intratracheal inoculation of a virulent strain of IBV.

Inoculum	2 WPI				3 WPI			
	UT	MT	LT	Average	UT	MT	LT	Average
MB	73.5 (20.7)^a^	54.0 (9.3)^a^	43.2 (7.5)^a^	56.9 (10.4)^a^	56.3 (13.9)^a^	46.0 (9.4)^a^	43.1 (6.2)^a^	50.1 (8.5)^a^
MS-H	79.0 (24.9)^a^	63.7 (16.8)^a^	45.6 (9.4)^a^	63.5 (12.3)^a^	67.1 (9.4)^a,b^	50.7 (11.2)^a^	44.9 (8.7)^a^	54.2 (7.8)^a^
AS2	76.1 (10.6)^a^	55.1 (6.0)^a^	52.3 (6.4)^a^	61.7 (7.0)^a^	79.6 (33.1)^a,b^	57.7 (12.9)^a^	50.2 (21.3)^a^	62.8 (20.3)^a,b^
AB1	82.6 (18.8)^a^	66.9 (22.4)^a^	49.7 (7.0)^a^	68.5 (18.3)^a^	110.2 (38.2)^a,b^	63.2 (20.6)^a^	52.9 (10.6)^a^	75.4 (20.4)^a,b^
TS4	131.5 (56.5)^b^	66.0 (10.5)^a^	58.5 (24.3)^a^	85.4 (26.0)^a^	126.0 (51.2)^c^	66.0 (13.3)^a^	58.8 (13.0)^a^	86.5 (26.4)^b^
7NS	92.8 (16.3)^a^	62.0 (16.9)^a^	56.5 (9.4)^a^	70.6 (7.6)^a^	91.3 (25. 1)^b^	50.7 (10.0)^a^	58.2 (21.5)^a^	66.7 (12.8)^a,b^

Data are presented as means (standard deviation). Statistically significant differences within each column are shown with different lowercase superscript letters, *p <* 0.01 (Tukey’s corrected 2-way ANOVA). WPI, weeks post inoculation; MB: mycoplasma broth. Average denotes the average figure calculated from mucosal thicknesses measured at three distinct levels of trachea, upper, middle, and lower.

## Discussion

In this study, we selected three MS-H reisolates containing reversions to wildtype 7NS solely in ObgE, OppF, or GAPDH proteins, in order to evaluate the effect of gradual combinations of these three sequence variations *in vivo*. Based on our recent observations, such genomic patterns may occur, albeit rarely, in vaccinated poultry flocks. The *in vivo* experiment in the present study was designed to examine an extreme scenario where such MS-H derivatives carrying sequence reversions may appear and circulate within a population of field chickens. Thus, the strains were administered by aerosol to the “inoculated” groups. These chickens were co-inoculated with a virulent strain of IBV to exacerbate the gross and microscopic lesions (Markham et al., 1998). In addition to this, “in-contact” chickens were added to each group to investigate pathogenicity and humoral response following horizontal transmission in chickens. It is notable that the strain of chickens used in this study (mini leghorn) may behave differently to those used in commercial production, and therefore future studies are needed to investigate the effect of these strains on the chickens used for poultry meat and/or egg production.

High seropositivity rates were observed in the TS4 and AB1 inoculated chickens 3 weeks after inoculation. More importantly, the humoral responses of the in-contact chickens in groups exposed to the MS-H reisolates were consistent with the high systemic antibody responses to *M. synoviae* occasionally observed in a vaccinated flock (Kordafshari et al., 2020). Notably, the only group of in-contact chickens which developed more severe lesions were the group in-contact with the 7NS inoculated chickens despite the low reisolation rate, serological response, and tracheal mucosal thickness in 7NS inoculated chickens. It is plausible that the fitness of the primary 7NS inoculum in chickens had somewhat decreased upon successive *in vitro* cultures and was regained after one passage *in vivo*. It is unclear whether this was due to sequence modifications or transient metabolic adaptations to these changing conditions. Future work to determine the whole genome sequence of the 7NS clones isolated from the in-contact chickens will be required to elucidate this question. The level of relative antibody units and the gross air sac lesions was not significantly different in the chickens in-contact with those inoculated with MS-H reisolates compared to that of chickens in-contact with MS-H inoculated chickens. These findings suggest that such regained fitness observed in wildtype 7NS did not occur in MS-H reisolates following passage *in vivo*. This is also consistent with the usual absence of serious pathology in vaccinated flocks, even when MS-H strains with some sequence reversions are isolated from the farms. While aerosol administration is not the registered vaccination method for MS-H, it was used to inoculate the chickens for consistency in reproducing the airborne propagation of *M. synoviae* which is expected to occur in a flock. The finding that the MS-H vaccine was not reisolated from the trachea following primary aerosol administration has been reported previously (Noormohammadi et al., 2003) and is likely due to a sub-optimal inoculation and/or dose.

### ObgE has a role in transmission but not in virulence

All the three reisolates assessed in this study carried the wildtype ObgE. This protein has a role in ribosome maturation, DNA replication initiation and chromosome segregation in *Caulobacter crescentus*, *Bacillus subtilis*, and *Escherichia coli* (Morimoto et al., 2002; Ulanowska et al., 2003; Datta et al., 2004; Kint et al., 2014) and is involved in the thermosensory mechanisms of *M. synoviae* (Shahid et al., 2013b). All the three reisolates containing variation in *obgE* showed a *ts^−^* phenotype which is consistent with previous reports on association of this variation in *obgE* and loss of *ts* phenotype in MS-H reisolates (Shahid et al., 2013b). Interestingly, despite the loss of *ts* phenotype in these reisolates *in vitro*, only the TS4 reisolate was recovered significantly higher than MS-H from the lower trachea of inoculated chickens 3-week post inoculation. This finding confirms that the capacity to grow at higher temperatures *in vitro* does not always exclude colonization in lower parts of the respiratory tract *in vivo*. In the in-contact chickens 3 weeks after the exposure, the AS2 reisolate was reisolated at a higher rate compared to other reisolates, suggesting transmission of AS2 from the inoculated chickens to a greater number of in-contact chickens. However, the absence of any significant air sac lesions in the in-contact chickens exposed to the AS2 reisolate suggest that the reversion in ObgE does not have an impact on pathogenicity following horizontal transmission. The AS2 reisolate showed a significantly higher lesion scores in gross air sac examination in inoculated chickens but was not isolated from air sacs. For consistency, a particular section of the left abdominal air sac was swabbed, but air sac lesions were more commonly detected in the right abdominal air sac, which may explain the low isolation rate. The increased air sac lesions observed in the AS2 inoculated chickens but not in the in-contact chickens exposed to the AS2 might be due to a higher dose of inoculum received after artificial aerosalisation compared to natural transmission.

### OppF is involved in humoral immune responses

The oligopeptide (Opp) permease in mycoplasmas consists of two integral membrane proteins, OppB and OppC, two nucleotide-binding proteins, OppD and OppF, and an extracellular substrate-binding protein, OppA (Zhu et al., 2019). The OppA subunit of *M. hominis* and the OppD subunit of *M. gallisepticum* have been reported to be involved in host cell attachment and colonization, respectively, suggesting the Opp permease is likely to play a role in virulence (Hopfe et al., 2011; Tseng et al., 2017). The reisolation and seropositivity rates of AB1 inoculated chickens were significantly higher than those of MS-H inoculated chickens 3 weeks after inoculation. A significant greater upper tracheal mucosal thickness was observed in the AB1 inoculated chickens compared to that of AS2 and MS-H inoculated chickens, as well as significant higher reisolation and seropositivity rates compared to those of MS-H inoculated chickens 3 weeks after inoculation. These findings lend support to the possibility of the role of OppF in the improved attachment or multiplication of *M. synoviae* onto host cells in the upper trachea and induction of the immune response. However, there was no significant difference in the lower and middle mucosal thicknesses and the air sac lesions of the AB1 inoculated chickens compared to those of MS-H and AS2 inoculated chickens. These findings imply that reisolates containing the wildtype OppF and ObgE are less likely to affect lower parts of the respiratory tract and therefore, less likely to lead to substantial clinical signs. The absence of any significant reisolation rate and air sac lesions in the in-contact chickens exposed to the AB1 inoculated chickens indicates that the concurrent reversions in ObgE and OppF do not affect pathogenicity following horizontal transmission in the poultry flocks.

### GAPDH is a key player in pathogenesis

The genome of *M. synoviae* 7NS strain contains two identical copies of the 1005 bp *gapdh* gene positioned on each side of the *vlhA* locus. However, these coding regions differ from each other (and from 7NS) in MS-H genome, with G to A SNPs at positions 554 of the MSH_RS01150 (GAPDH-1150) and 917 of the MSH_RS01365 (GAPDH-1365) genes (Zhu et al., 2019). The GAPDH-1150 of TS4 isolate is identical to that of MS-H, but the GAPDH-1365 is identical to GAPDH of 7NS. The homologous GAPDH protein in many mycoplasmas has been shown to contain a surface localized fraction contributing to host cell adhesion, with the C-terminal region confirmed in the cytoadhesion of *M. pneumoniae* (Alvarez et al., 2003; Dumke et al., 2011; Gründel et al., 2016; Song et al., 2018; Grimmer and Dumke, 2019; Wang et al., 2021). Both copies of GAPDH in TS4 reisolate contain the identical C-terminal to that of 7NS. Among the reisolates, the TS4 reisolates showed the highest reisolation and seropositivity rates as well as the greatest mucosal thicknesses in upper trachea. These findings indicate the possibility of an improved attachment or multiplication of TS4 onto host cells in the upper trachea and induction of the immune response due to the presence of wildtype GAPDH-1365. While TS4 reisolate showed a superior pathogenicity in the upper trachea, the absence of any significant differences in middle and lower trachea indicates the virulence of this reisolate is less likely to be enhanced drastically. The incremental increase in pathogenicity and immunogenicity of TS4 compared to those of AB1, and of AB1 compared to those of AS2 suggest a cumulative effect of the wildtype *obgE*, *oppF* and *gapdh* genotypes. However, the probability of occurrence of concurrent reversions is likely to be negligible at a flock level. Furthermore, the developed immunity following vaccination in the majority of chickens in poultry flocks provides protection against such reisolates. Since the AS2, AB1 and TS4 reisolates emerged in vaccinated populations, a further study involving challenging vaccinated SPF chickens or field chickens with these reisolates would provide a more realistic picture of their significance in the field.

In conclusion, this study demonstrates that while the MS-H reisolates (even containing three wildtype proteins) might induce a higher systemic antibody response, they do not cause pathogenicity in lower parts of the respiratory system, and thus less likely to result in significant clinical signs. The study provides important insights into the molecular basis of attenuation of the MS-H vaccine and emphasizes the role of ObgE, GAPDH and OppF proteins in pathogenicity, immunogenicity, and transmission of *M. synoviae*. These results also have implications for Mycoplasma species affecting other animals and humans.

## Data availability statement

The data presented in this study have been deposited in the NCBI GenBank repository under accession numbers CP103982 (AS2), CP103981 (AB1), and CP103980 (TS4).

## Ethics statement

The animal study was reviewed and approved by the University of Melbourne Animal Ethics Committee under approval number 21064.

## Author contributions

SK: substantial contributions to the design of the work, and the acquisition, analysis, interpretation of data for the work. Drafting the work OO, SZ, PA, PV, PS, NW, AC, DO, JD, and DA: substantial contributions to the acquisition of data for the work and revising the work critically for important intellectual content. GU, CM, MM, and AN: substantial contributions to the conception and design of the work and the interpretation of data for the work and revising the work critically for important intellectual content. All authors contributed to the article and approved the submitted version.

## Funding

This study was funded by the Australian Research Council and Bioproperties Pty. Ltd. through the ARC Linkage Project grant LP180100762.

## Conflict of interest

This study was funded by the Australian Research Council and Bioproperties Pty. Ltd. through the ARC Linkage Project grant LP180100762.

The MS-H vaccine is produced by the Bioproperties Pty. Ltd., Australia. The authors declare that this study received funding from Bioproperties Pty. Ltd. The funder had the following involvement in the study: study design, decision to publish, and reviewing the manuscript.

## Publisher’s note

All claims expressed in this article are solely those of the authors and do not necessarily represent those of their affiliated organizations, or those of the publisher, the editors and the reviewers. Any product that may be evaluated in this article, or claim that may be made by its manufacturer, is not guaranteed or endorsed by the publisher.
