# Supplementary material for: Virulence factors of Mycoplasma synoviae: Three genes influencing colonization, immunogenicity, and transmissibility
Source: Front Microbiol. 2022 Nov 25;13:1042212. doi: 10.3389/fmicb.2022.1042212 (PMC9749132; doi:10.3389/fmicb.2022.1042212)
Supplement: Supplementary file 1 [file Table_1.DOCX]

| **Table S1.** Scores from the gross air sac lesions, two and three weeks after aerosol inoculation of SPF chickens with various strains or reisolates of MS following intratracheal inoculation with IBV, and two and three weeks after exposure of in-contact chickens with the inoculated chickens | | | | | | | |
| --- | --- | --- | --- | --- | --- | --- | --- |
|  | **Inoculated birds** | | |  | **In-contact birds** | | |
| **Inoculum** | **2WPI** |  | **3WPI** |  | **2WPE** |  | **3WPE** |
|  |  |  |  |  |  |  |  |
| MB | 0 (1 – 0)^a^ |  | 0 (1 – 0)^a^ |  | 0 (0 – 0)^a^ |  | 0 (0 – 0)^a^ |
| MS-H | 0.75 (1 – 0)^ab^ |  | 1.25 (4 – 0)^a^ |  | 0 (0 – 0)^a^ |  | 0 (1.5 – 0)^a^ |
| AS2 | 2.25 (10 – 0)^b^ |  | 0.75 (4 – 0)^a^ |  | 1 (3 – 0)^a^ |  | 0 (2 – 0)^a^ |
| AB1 | 1.75 (5.5 – 0.5)^ab^ |  | 1.5 (3 – 0)^a^ |  | 0 (1.5 – 0)^a^ |  | 1 (1 – 0)^a^ |
| TS4 | 1.5 (6 – 0)^ab^ |  | 1.5 (3.5 – 0)^a^ |  | 0 (0 – 0)^a^ |  | 0 (1 – 0)^a^ |
| 7NS | 2 (5.5 – 0)^b^ |  | 0.75 (5 – 0)^a^ |  | 0 (1 – 0)^a^ |  | 0 (11 – 0)^a^ |
|  |  |  |  |  |  |  |  |

Data are presented as median (range). Statistically significant differences within air sac lesion scores are shown with different lowercase superscript letters, *p ˂* 0.01 (Dunn’s corrected Kruskal-Wallis test). Air sac lesions were scored grossly for severity on a scale of 0 to 3, and a cumulative score was determined for each bird by adding the scores of all air sacs. WPI, weeks post inoculation. WPE, weeks post exposure.
