# Supplementary material for: Virulence factors of Mycoplasma synoviae: Three genes influencing colonization, immunogenicity, and transmissibility
Source: Front Microbiol. 2022 Nov 25;13:1042212. doi: 10.3389/fmicb.2022.1042212 (PMC9749132; doi:10.3389/fmicb.2022.1042212)
Supplement: Supplementary file 2 [file Table_2.DOCX]

| **Table S2.** Titres of cultures used for aerosol inoculation | | |
| --- | --- | --- |
| **Inoculum** | **Initial titre (CCU)/mL** | **Volume of culture delivered in the infection chamber (mL)** |
| MS-H | 1.69 × 10^7^ | 36 |
| AS2 | 1.18 × 10^7^ | 34 |
| AB1 | 1.69 × 10^7^ | 35 |
| TS4 | 3.66 × 10^7^ | 32 |
| 7NS | 3.49 × 10^7^ | 34 |

AS2, MS-H reisolate containing reversion in *obgE* gene; AB1, MS-H reisolate containing reversions in *obgE* and *oppF* genes; TS4, MS-H reisolate containing reversions in *obgE*, *oppF* and *gapdh* genes.
