# Supplementary material for: Virulence factors of Mycoplasma synoviae: Three genes influencing colonization, immunogenicity, and transmissibility
Source: Front Microbiol. 2022 Nov 25;13:1042212. doi: 10.3389/fmicb.2022.1042212 (PMC9749132; doi:10.3389/fmicb.2022.1042212)
Supplement: Supplementary file 3 [file Table_3.DOCX]

| **Table S3.** Relative antibody units against the MSPB region of the *vlhA* gene, two and three weeks after inoculation of SPF chickens with various strains or reisolates of MS following intratracheal inoculation with IBV, and two and three weeks after exposure of in-contact chickens with the inoculated chickens | | | | | |
| --- | --- | --- | --- | --- | --- |
| **Inoculum** | **Inoculated birds** | |  | **In-contact birds** | |
|  | **2 WPI** | **3WPI** |  | **2 WPE** | **3 WPE** |
| MB | 2.15 (2.3)^a^ | 2.75 (1.53)^ab^ |  | 3.77 (1.32)^a^ | 8.5 (3.54)^ab^ |
| MS-H | 6.74 (6.02)^a^ | 13.62 (8.91)^ab^ |  | 55.32 (113.34)^a^ | 20.56 (20.01)^ab^ |
| AS2 | 19.32 (18.92)^a^ | 65.23 (66.65)^abc^ |  | 36.46 (50.27)^a^ | 380 (103.3)^cd^ |
| AB1 | 52.57 (50.2)^a^ | 317.94 (277.7)^bc^ |  | 25.33 (39.19)^a^ | 108.2 (163.15)^abd^ |
| TS4 | 656.15 (437.18)^b^ | 687.58 (396.28)^d^ |  | 34.23 (41.49)^a^ | 45.22 (83.32)^abd^ |
| 7NS | 46.06 (88.69)^a^ | 170.92 (156.27)^abc^ |  | 18.83 (21.49)^a^ | 223.69 (220.05)^cbd^ |

Data are presented as means (standard deviation). Statistically significant differences within each column are shown with different lowercase superscript letters, *p ˂* 0.01 (Tukey’s corrected 2-way ANOVA). WPI, weeks post inoculation. WPE, weeks post exposure.
