# Supplementary material for: Virulence factors of Mycoplasma synoviae: Three genes influencing colonization, immunogenicity, and transmissibility
Source: Front Microbiol. 2022 Nov 25;13:1042212. doi: 10.3389/fmicb.2022.1042212 (PMC9749132; doi:10.3389/fmicb.2022.1042212)
Supplement: Supplementary file 4 [file Data_Sheet_1.PDF]

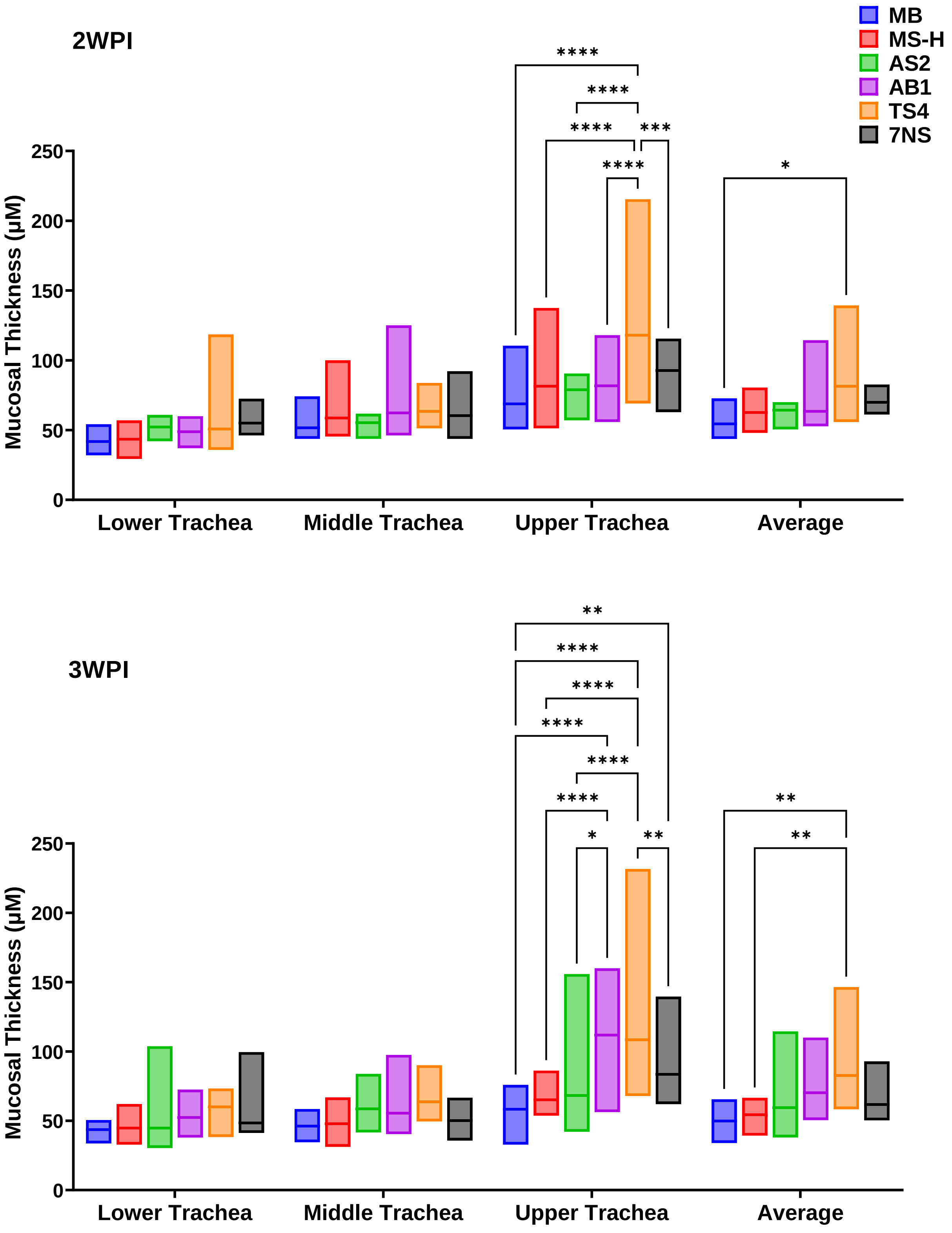
2WPI
MB
MS-H
AS2
AB1
TS4
7NS
Mucosal Thickness (µM)
0
50
100
150
200
250
****
****
****
***
****
*
Lower Trachea
Middle Trachea
Upper Trachea
Average
3WPI
Mucosal Thickness (µM)
0
50
100
150
200
250
**
****
****
****
****
****
*
**
**
**
Lower Trachea
Middle Trachea
Upper Trachea
Average

Figure S1. Mucosal thicknesses of upper trachea (UT), middle trachea (MT), and lower trachea (LT) (floating bars showing the data range and line indicating the mean value) collected two and three weeks after inoculation of test chickens with various strains or reisolates of *M. synoviae* following intratracheal inoculation with a virulent strain of IBV on the top, and two and three weeks after exposure of in-contact chickens to inoculated birds on the bottom. Average denotes the average figure calculated from mucosal thicknesses measured at three distinct levels of trachea, upper, middle, and lower. *, $p < 0.05$. **, $p < 0.01$. ***, $p < 0.001$. ****, $p < 0.0001$ (Tukey's corrected 2-way ANOVA test). WPI, weeks post inoculation. AS2, MS-H reisolate containing reversion in *obgE* gene; AB1, MS-H reisolate containing reversions in *obgE* and *oppF* genes; TS4, MS-H reisolate containing reversions in *obgE, oppF* and *gapdh* genes.
